# Use of a Sensing Device to Visualizes Group Participation in Social Skills Learning Groups

**DOI:** 10.3389/fpsyt.2021.642949

**Published:** 2021-04-13

**Authors:** Tomoka Yamamoto, Hiroko Okuno, Aika Tatsumi, Saeko Sakai, Ikuko Mohri, Masako Taniike

**Affiliations:** ^1^Molecular Research Center for Children's Mental Development, United Graduate School of Child Development, Osaka University, Suita, Japan; ^2^United Graduate School of Child Development, Osaka University, Suita, Japan; ^3^Research Development, Hyogo Institute for Traumatic Stress, Kobe, Japan

**Keywords:** autism spectrum disorder, social skills intervention, group participation, visualizing behaviors, social network

## Abstract

Children with autism spectrum disorders (ASD) have difficulties in developing stable peer relationships. Interventions for learning social skills (SS) for such children are often conducted in a group. Behavioral imaging and social imaging, which have been called for in recent years, are methods for visualizing children's behaviors and interpersonal relationships. To examine the usefulness of visualizing face-to-face interaction with others in the social skills learning scene of children with ASD, we use a business microscope that can qualify and visualize face-to-face interactions automatically. We highlight two boys' face-to-face interaction changes in the same SS learning group of five children. The device's use may provide a more objective measurement that complements the observer's subjective evaluation in case of the intervention's validation. It is expected that information on face-to-face interactions will be used to determine the SS learning process in the future.

## Introduction

Autism spectrum disorders (ASD) are characterized by difficulty in communicating and interacting in social settings and limited and repetitive behaviors, interests, and activities (1). Children with ASD may face challenges in group interactions (e.g., while they spend time with others from the same age group) at school. Regarding the peer relationships of children with ASD, it has been observed that such children are often involved only at the periphery and not at the center of a class, they tend to be a part of a relatively small group, and they lack reciprocity (2).

It has been established that one of the factors influencing the formation of peer relationships is the development of social skills (SS); however, children with ASD have difficulty learning such skills (3). Interventions for learning SS, such as SS training for school-aged children with ASD, have thus been implemented (4). Specifically, interventions for SS learning, based on cognitive behavioral therapy and social learning theory (5), often consist of structured classes related to specific skills, modeling skills, role-playing and rehearsing skills, and offering feedback on children's role-play/performance (6). In addition to effects such as improved social competence (6) and increased prosocial behavior [(7), etc.], psychological effects, such as improvements in (or development of) actual friendships (6), decreased depression and anxiety (8), and improved mental health (9), have been observed. Therefore, SS learning programs in groups (i.e., Group Social Skills Intervention; GSSI) are often implemented (10).

In addition to practicing skills with multiple partners and groups, which is effective in SS teaching (11), the importance of relationships and connections within groups in group-based SS learning has been highlighted (12). For this reason, some GSSI group studies have attempted to identify changes in the approaches of children with ASD toward other children through behavioral observation methods. However, this approach is not practical since these behavioral observations are rarely conducted in a blind manner (7).

## Behavioral Imaging and Social Imaging

In recent years, attempts have been made to visualize children's behavior with ASD using various technologies. Behavioral imaging comprises computational sensing and modeling technology, used to measure, analyze, and understand human behavior, and provides a quantitative understanding of human behavior and development. Imaging technologies to monitor these behaviors and social interactions are diverse and include video, audio, and sensing technologies such as body sensors and location-based systems (13). Such technologies have been used to diagnose and assess ASD and help with the intervention (13). Furthermore, Suzuki (14), focusing on interpersonal interactions specifically, proposed social imaging as a method for visualizing interactions between several people. They created a device that measures and enhances interpersonal interactions by wearing a headband that glows when face-to-face interactions occur. (15). In GSSI, these technologies can also be applied to quantify and visualize the relationship between children with ASD and the group(s), using the obtained knowledge to provide intervention in learning situations.

Business microscopes (herein, BMS, (16)) are sensing devices that can quantify communication with others. This device can be worn on the body naturally like the nametag. The main components of the sensor include infrared transmission/reception systems and built-in 3D activity trackers. The infrared sensor detects face-to-face events at a distance of 2–3 m and within a 120-degree ° angle, either right or left. The person facing the device was recorded. It should be noted that the device used has a built-in tri-axis accelerometer that records the wearer's physical activity in Hz. The infrared sensor data and physical activity data were sent to the Human Big Data Cloud Service and analyzed. The analyzed data can be downloaded from the server by specifying the time and format (e.g., the time series CSV data about face-to-face partner and physical activity, the value calculated by the time series CSV data, and the network diagram). The type of communication can be estimated by physical movements (any physical movement with 2.0 Hz or more was defined as an active state, while any movement measuring <2.0 Hz was defined as a non-active state) and face-to-face interactions automatically.

When there was face-to-face interaction where the participant was active and the other party was non-active, the participant was defined as the “pitcher” of the communication (e.g., talking). Conversely, if the participant was non-active, but the other party was active, the participant was described as the “catcher” of the communication. If both parties were active, the communication was defined as “two-way.” If both parties were non-active, they were deemed “face-to-face.” Additionally, a network diagram can be drawn from the measured data automatically based on the face-to-face time between participants. In this diagram, the edge connecting the nodes (i.e., each person) appears thicker as the face-to-face time increases.

Although such diagrams were initially developed to visualize communication and improve productivity in business [e.g., performance in call centers (17)], in recent years, research regarding the visualization of communication has been conducted concerning pedagogy and developmental psychology (18). Efforts have also been made to use these diagrams to better understand changes associated with development in groups (18) and improve classes for teachers in physical education (19). It has been suggested that the real-time visualization of communication can provide an objective view for childcare workers and teachers. However, few to no studies have been conducted on children with ASD who have difficulty with social interaction. Therefore, it is necessary to examine the adaptation of children with ASD within a group of peers.

## The Pilot Study About Measuring Children's Behavior Change by BMS

**Figure 1:**
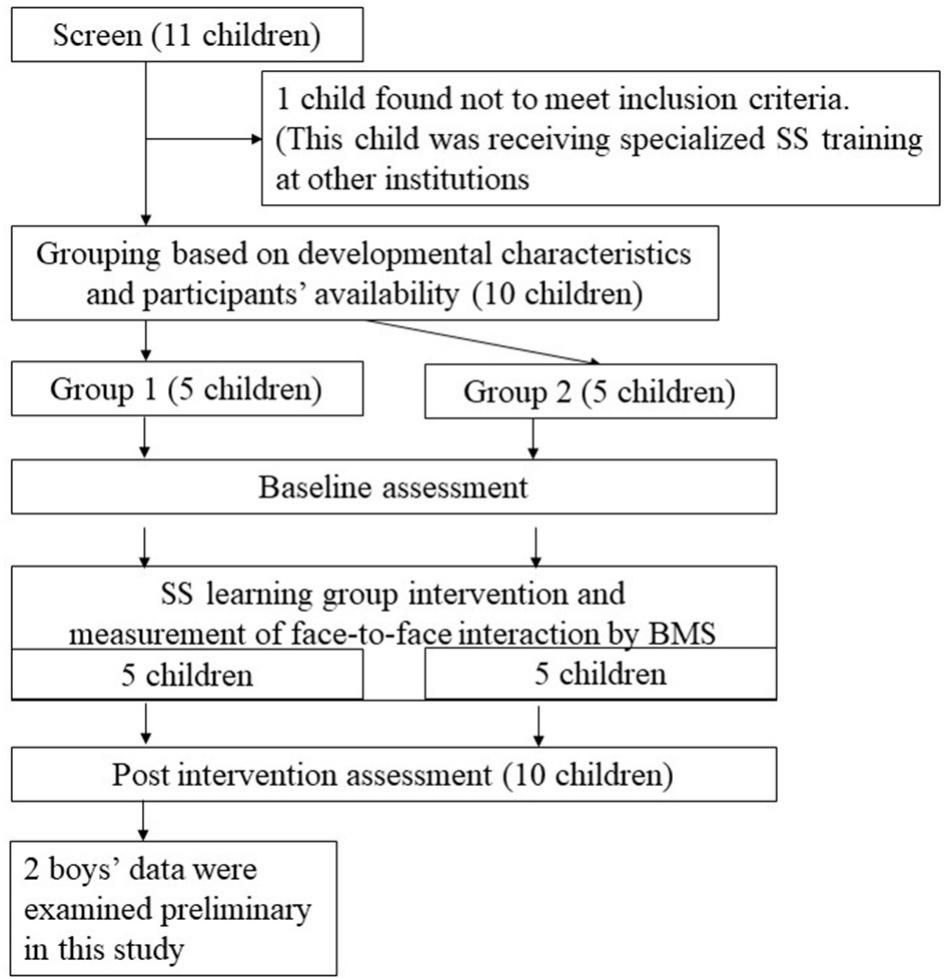
Flow diagram showing enrollment and study design.

In order to examine the effectiveness of visualizing face-to-face interaction in SS learning groups, the data of face-to-face interaction during free playtime in the SS learning group were collected by BMS. Five children with ASD, ranging from third to fourth-grade elementary students, participated in this study's SS learning program. Children had been under medical examination as part of their developmental disorder outpatient treatment. According to the Diagnostic and Statistical Manual of Mental Disorders, all participants were diagnosed (1). Given our study's preliminary stage, we will highlight two boys, Boy A (fourth grade) and Boy B (third grade) (Figure 1).

The STSSE (20) was modified to create a program that taught verbal communication skills (e.g., listen, explain, converse, and negotiate) to participants for a total of 10 sessions. Each session lasted about an hour and a half and included teaching, modeling, role-playing, rehearsing, feedback, and homework related to the target skills. Aside from the five children who took part in the program, a trainer and sub-trainer responsible for teaching the program and three clinical psychology students participated in this study as staff members. On the same day as the children's respective sessions, their caregivers participated in a program to encourage their children to extend the SS they had learned during each session to their home and school environments. This program was created by modifying the STSSE. The parents' sessions comprised group discussion, role-play, watching a videotaped recording of their children's sessions, and weekly homework assignments.

In this study, a mat was laid at the back of the room during the allocated free playtime. Further, toys for playing games with rules, toys for playing with imagination, construction toys, toys with a cause and effect, and cushions were placed on the mat. After arriving at the room, the participants and trainers began each session by hanging their BMS devices around their necks. Children were explained BMS device as the nametag and hanged it through the GSSI sessions. Each device was customized with participants/trainers' names. None of the participants had a problem related to wearing the BMS devices.

Changes in the type of face-to-face communication were measured every 2.5 s to capture the changes in the face-to-face behavior (i.e., face-to-face interaction partner). This was performed in both the first and ninth sessions (in the first session, a free play activity time was held for the first time, and in the ninth session, a free play activity time was held for the last time) between the participants and the person they were facing. A face-to-face network diagram was also drawn. A network diagram was drawn using the free play activity scene's behavior to measure this behavior in a natural setting, in which the staff provided the least prompts. In this study, Cytoscape 3.8.0 was used for visual clarity. The edge connecting the nodes (i.e., each person) has a thickness of five levels—the longer the face-to-face time, the thicker and darker the color appears in this diagram. The network diagrams of the first and ninth network sessions were then compared. The first free-play activity scene was 16 min long, and the ninth free-play activity scene was 15 min long.

Supplementary, the child's classroom teacher completed the Social Skills Scale (herein, “SS scale”; (21)) at two-time points to understand the acquisition of SS before and after the intervention. The SS Scale is an itemized questionnaire rated by those who can directly observe children's behavior in a group setting. This scale comprises four areas: group behavior skills, communication skills, self-control skills, and peer relationship skills. The mean of the standard scores was 10 (1**–**15).

**Figure 2:**
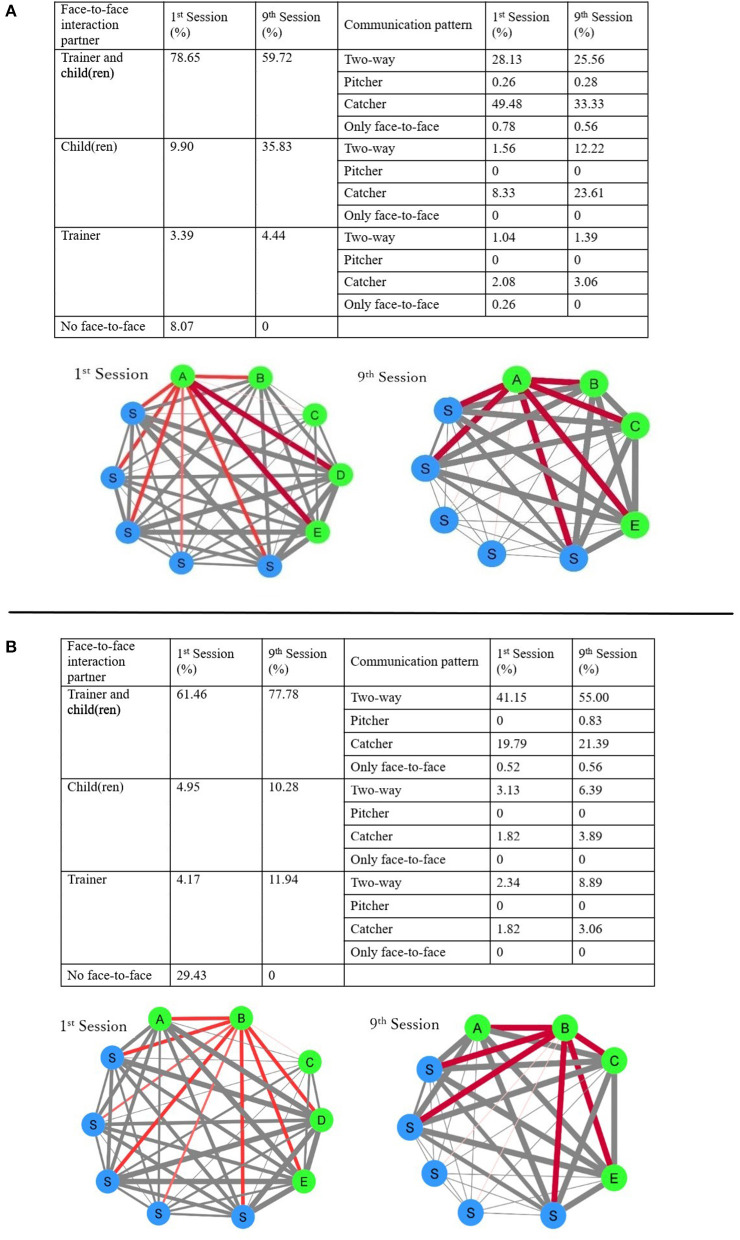
Boy **(A)** changed of percentages that participants faced people and percentage of their communication pattern (above) and the network diagrams (below). Boy **(B)** changed of percentages that participants faced people and percentage of their communication pattern (above) and the network diagrams (below). The thinnest line in the network diagram indicates face-to-face interaction was the least, while the thickest line indicates that face-to-face interaction was the most.

Boy A: Boy A, 9 years 9 months old, is in the fourth grade of elementary school. He has a full-scale IQ (FSIQ) of 80, with a comprehensive verbal index of 72, perceptual reasoning index of 95, working memory index of 82, processing speed index of 86. Boy A fulfilled the criteria for ASD and ADHD. He scored 63 on the Social Responses scale (Awareness 52, Cognition 68, Communication 55, Motivation 70, and Mannerism 63). As a toddler, he had difficulties communicating (e.g., language delay, immediate echolalia), little interest in other children, obsession, and restricted interest in one character. Although he was not administrated Autism Diagnosis Observation Scale (ADOS), his severity of autism was estimated as mild based on his symptoms and developmental history. When starting GSSI sessions, his obsession had decreased, but difficulties of asking for help and challenges of group participation had persisted. The parents reported about his friendship, “It is unclear to me as to whether or not he interacts with others.” He participated in nine of ten GSSI sessions. He demonstrated a change in the percentage of time when he was not facing anyone through GSSI sessions (8.1% in the first session and 0% in the ninth session). Conversely, the time he spent with other children increased from 9.9% in the first session to 35.8% in the ninth session (Figure 2A). Concerning the type of communication, Boy A, regardless of the face-to-face interaction partner, remained less likely to engage in a voluntary approach (i.e., being the pitcher in the communication). However, there was an increase in his two-way communication with other children (1st 1.56% → 9th 12.22%) and in cases where other children approached him (Catcher 1st 8.33% → 9th 23.61%). As seen in the network diagram, D and E's network was strong in the first session. There was little interaction with B and C. Furthermore, his network with the staff members, who mainly participated in the free play activities, was narrow. In the ninth session, Boy A's network among other children was strong, and his interaction with the three staff members, who were mainly involved in the free play activities, was also strong. However, Boy A did not show more interaction with specific children (Figure 2A). On the SS-scale, the skills that changed by more than 1 standard deviation were: group behavior (7 points → 13 points) and peer relationships (7 points → 14 points)

Boy B: Boy B, 8 years 4 months old, is in the third grade of elementary school. He has a full-scale IQ (FSIQ) of 105, with a comprehensive verbal index of 123, perceptual reasoning index of 106, working memory index of 100, processing speed index of 78. He scored 83 on the Social Responses scale (Awareness 49, Cognition 76, Communication 79, Motivation 75, and Mannerism 103). Boy B fulfilled the criteria for ASD and ADHD. As a toddler, he lacked interest in others and obsessed with orders, restricted interest in the train, and sensory sensibility. At the time of starting GSSI sessions, he had little interest in others, difficulties in conversation, restricted interest. He was administrated ADOS at 7 years, his severity of autism was mild. The parents stated, “He looks like he has no interest in other children.” He participated in seven of ten GSSI sessions. He demonstrated a change in the percentage of time when he was not facing anyone (1st 29.4% → 9th 0%). The time he spent with other children increased from 5.0% in the first session to 10.3% in the ninth session. Furthermore, his two-way communication increased regardless of the face-to-face interaction partner (1st 46.61% → 9th 70.28%). There is evidence that he had little to no interaction with the staff and other children in the first session in the network diagram but started showing a network line with maximum thickness in the ninth session (Figure 2B). He only showed changes in peer relationships (8 points → 12 points) at the SS-scale.

## Discussion

This study attempted to visualize and quantify communication in a group of SS learning programs using a business microscope.

In terms of face-to-face interaction partners, in the free play activity scene, the time that the participants did not spend with anyone decreased, and the time they spent with other children increased. Concerning the type of communication, although there was no significant change in Boy A's voluntary approach (i.e., toward becoming a communication pitcher), he experienced increased occurrences of being approached by other children. Furthermore, Boy B showed an increase in his two-way communication, regardless of whom he was communicating with. In the video footage of their behavior, both Boy A and Boy B were seen taking part in a face-to-face play with staff members in the first session, engaging in cooperative play, and social play with simple rules with other children in the eighth session. Changes in face-to-face interaction partners and their communication types are believed to reflect their behavioral differences. It has been noted that children's appropriate social approaches within groups increase when they are provided with opportunities to learn SS (7, 22). The current study further supports this previous finding.

Further, the encouragement of participating staff to interact with other children helped them gain necessary social skills. There was also an increase in the participants' peer relationship skills at school. As observed in the BMS, it is presumed that such improvements would also be observed in their school settings.

The observer's issue knowing that intervention occurs during behavioral observations in SS groups for children with ASD has already been highlighted (23). To address this issue, in this study, although behavior observation was performed using a business microscope, the observation would be useful in the sense that the viewpoint of the observer was not included since the use of a sensor device allowed for an objective evaluation. The use of the device is potentially being a more objective measurement which complements the subjective evaluation.

Moreover, the utilization of a sensor device that visualizes interpersonal information in GSSI allows for the easy identification of detailed information such as interactions with multiple people. Thus, immediate feedback of intervention results may facilitate intervention policy modification (13). Nakajima et al. (24) conducted an initiative that involved middle school teachers who conducted physical education classes.

The authors showed them a network diagram, gave feedback on verbal communication, and improved their physical education classes. Similarly, network diagrams for the training of trainers/caregivers of autistic children may lead to appropriate interventions for children with ASD who tend to have difficulty in dealing with people.

However, BMS cannot be used immediately in clinical situations. Although BMS data can be used to evaluate quantifiable changes, such as the amount of body movement or a person with whom one comes into face-to-face contact, it is challenging to evaluate the noted behavior's social suitability. In the future, it will be necessary to enable the objective evaluation of the content of approaches by combining the BMS with other devices that allow for the analysis of the content of the communication. Furthermore, for evidence-based evaluation, it is essential to examine the relationship between face-to-face communication and children's skills enhancement. However, only two cases were considered, and statistical analysis was absent in this study. Therefore, larger sample sizes and data from different types of children with ASD should be considered.

Furthermore, although many SST trainers may feel that the relationship between children in the group improves growth, it has not been examined quantitatively. In the future, quantifying face-to-face interaction by BMS enables us to determine how interactions with others affect social skills learning.

## Data Availability Statement

The raw data supporting the conclusions of this article will be made available by the authors, without undue reservation.

## Ethics Statement

The studies involving human participants were reviewed and approved by Osaka University Clinical Research Review Committee. Written informed consent to participate in this study was provided by the participants' legal guardian/next of kin. Written informed consent was obtained from the minor(s)' legal guardian/next of kin for the publication of any potentially identifiable images or data included in this article.

## Author Contributions

TY: study design, data collection, data analysis, and writing manuscript. HO: data collection and writing manuscript. AT: study design and data collection. IM: data collection and writing manuscript. MT: writing manuscript. All authors contributed to the article and approved the submitted version.

## Conflict of Interest

The authors declare that the research was conducted in the absence of any commercial or financial relationships that could be construed as a potential conflict of interest.
